# Assessment of the Awareness of Pre-anesthesia Evaluations Among Healthcare Beneficiaries in a Tertiary Teaching Hospital in Central India: A Questionnaire-Based Survey

**DOI:** 10.7759/cureus.83255

**Published:** 2025-04-30

**Authors:** Ashutosh Kumar, Barkha D Agrawal, Prakash G Gondode, Omshubham Asai, Avinash Prakash, Bhuvaneswari Balasubramanian

**Affiliations:** 1 Department of Anaesthesiology And Critical Care, All India Institute of Medical Sciences Nagpur, Nagpur, IND; 2 Department of Anaesthesiology, Pain Medicine And Critical Care, All India Institute of Medical Sciences, New Delhi, Delhi, IND

**Keywords:** anesthesia, cross-sectional studies, general, health disparities, medical misconceptions, patient education, patient knowledge, post-pandemic, preoperative care, surgeons

## Abstract

Background

Pre-anesthesia evaluations (PAEs) play a crucial role in enhancing the safety of surgical procedures. This study aimed to assess the patients' understanding of PAEs in a tertiary care teaching hospital in Central India, identifying knowledge gaps and demographic disparities.

Methods

A cross-sectional study involving 413 patients and their attendants, attending the pre-admission anesthesia consultation (PAC) clinic, was conducted. The attendants were also included as they often play a vital role in decision-making and communication, particularly in the case of elderly or illiterate patients. This allowed us to capture the broader awareness of PAEs among healthcare beneficiaries who influence patient care. A questionnaire assessing 17 aspects of PAEs was administered. Data were analyzed based on demographics, education, and previous visits to the PAC clinic. The questionnaire was administered in multiple languages, and assistance was provided to illiterate patients to ensure accurate completion. Trained staff read questions aloud in the participant's preferred language (Hindi, Marathi, or English), explained key terms as needed, and ensured comprehension without guiding responses. This approach was standardized and approved as part of the study’s ethical protocol.

Results

Only 124 (30%) of the participants correctly understood the purpose of the PAE, while 201 (48.6%) believed it was necessary only for surgeries under anesthesia. This response reflects a common misconception. While it is true that PAE is not needed if no anesthesia is to be administered, it is important for procedures involving both general anesthesia and regional or monitored anesthesia care. Urban residents and those with previous PAC clinic visits demonstrated higher knowledge scores. Illiterate individuals scored lower than their educated counterparts, with significant differences across educational levels and age groups.

Conclusion

Although definitive large-scale data remains sparse, extant literature implies that the absence of complete PAEs may contribute to greater perioperative problems. PAEs are essential for perioperative planning and risk mitigation. This study emphasizes the need for more patient education and tailored interventions to understand PAEs. Clear communication and continual training for healthcare providers are essential for patient comprehension, engagement, and safer surgical techniques.

## Introduction

The role of anesthesiologists extends beyond administering anesthesia during surgery. They conduct an initial pre-operative assessment to identify existing medical conditions and evaluate perioperative risks. While they may order basic investigations or initiate referrals, the definitive management of comorbidities is typically carried out by appropriate specialists. The pre-admission anesthesia consultation (PAC) also serves as a setting to address patient anxieties through basic counseling and information-sharing; however, the extent and success of such interventions can vary. Although these efforts aim to reduce perioperative risk, outcomes depend on multiple factors and cannot be guaranteed. Furthermore, anesthesiologists explain the perioperative process, plan post-operative care, and, if necessary, advise on the postponement of surgeries to ensure patient safety. Their expertise in conducting a pre-anesthesia evaluation (PAE) contributes to enhancing operating room efficiency, minimizing surgical delays, and improving overall quality of patient care [1]. While PAEs are not capable of ensuring absolute safety, they are instrumental in identifying and mitigating risks, thus aiming to enhance surgical safety and patient outcomes.

With the growing trend of daycare surgeries and expanding healthcare access, PAEs are likely to play an increasingly important role in supporting surgical planning and patient safety, although further research is needed to validate their long-term impact. In the context of adult surgical patients, a well-conducted PAE is facilitated by patient awareness and understanding, which can improve communication, consent, and compliance with perioperative recommendations. Studies have shown that insufficient knowledge about PAEs exists across both developed and developing nations [2-4].

While studies on knowledge regarding PAEs have been conducted in various regions, few have focused on Central India, a region with distinct healthcare challenges. This region was selected for the study due to its unique healthcare challenges, including a large rural population with limited access to specialized healthcare services. Many patients from this area also have lower educational levels, which may affect their understanding of medical procedures, including the importance and process of PAEs. The differences in healthcare access, education, and awareness in this region present an important area for research, as understanding how patients in these regions perceive and engage with the PAE process is crucial for improving patient safety and healthcare outcomes. Given these challenges, this study aims to assess the knowledge and understanding of PAEs among patients in Central India.

## Materials and methods

Study setting and design

This study was conducted at the All India Institute of Medical Sciences Nagpur, a tertiary care teaching hospital in Nagpur, Maharashtra, India. The hospital serves a lower-to-middle-income population, with a significant proportion of patients from rural areas. Many of these patients have limited access to specialized healthcare, which can impact their health literacy and understanding of medical procedures, including PAEs. The study aimed to assess how demographic factors such as education, socio-economic status, and healthcare access influence patient knowledge of PAEs and to determine the need for targeted educational interventions.

A cross-sectional survey was conducted involving 413 patients and their attendants, attending the PAC clinic. A 17-question questionnaire (consisting of 12 knowledge assessment questions and five demographic questions) was used to evaluate patient knowledge, focusing on their understanding of the purpose and importance of PAEs, the role of the anesthesiologist, and the risks associated with anesthesia.

The questionnaire was administered in multiple languages, including Hindi, Marathi, and English. To ensure the inclusion of illiterate participants, trained staff provided standardized assistance. These staff members underwent a structured orientation led by the study team, covering research ethics, neutral communication techniques, and methods to confirm participant comprehension without influencing responses. They read each question aloud in the participant's preferred language and used standardized, non-leading explanations to clarify terms when necessary. The participant's understanding was confirmed through feedback, and the entire approach followed a protocol approved by the institutional ethics committee as a part of the informed consent process.

Responses were scored based on their consistency with current perioperative guidelines and best practices. The scoring key was reviewed and validated by a panel of senior anesthesiologists to ensure clinical relevance, contextual appropriateness, and alignment with standard protocols for PAE. No monetary or material incentives were involved in the scoring or participation process. The most clinically appropriate answer was scored as one mark, while incorrect or less appropriate responses were scored as zero. The questionnaire was adapted from the tool developed by Singla et al. [2]. While not a formally standardized instrument, this tool has been used in similar healthcare settings in India. To enhance validity, the tool was reviewed by a panel of senior anesthesiologists and pilot-tested for clarity and contextual suitability before its full-scale deployment.

Questionnaire details

The questionnaire assessed the patient's knowledge across several key areas of the PAE process. The questions were structured to evaluate the following aspects:

Purpose of the PAC Visit

What is the primary purpose of visiting the pre-admission anesthesia consultation (PAC) clinic?

Is the PAC visit necessary only for surgeries requiring anesthesia?

Role of the Anesthesiologist

Who is responsible for conducting the PAE?

What role does the anesthesiologist play in assessing and managing pre-existing medical conditions before surgery?

Pre-existing Medical Conditions

Which medical conditions should be disclosed to the anesthesiologist before surgery?

Why is it important to disclose conditions such as heart disease, renal problems, and respiratory issues?

Optimizing Health for Surgery

What steps are taken in the PAE to optimize the patient’s health before surgery?

How does the PAE contribute to the overall preparation for surgery?

Risks of Anesthesia

How do smoking, alcohol use, and other lifestyle factors affect anesthesia and surgery outcomes?

What are the potential risks of anesthesia that should be communicated to the anesthesiologist?

Perioperative Pain Management

Who is responsible for managing perioperative pain during and after surgery?

How does the anesthesiologist contribute to managing pain during the perioperative period?

The questionnaire consisted of 17 multiple-choice questions. Each question had one correct answer, but for some questions, multiple answers could be valid depending on the patient's perspective or clinical practice. The questions were presented in a simple, accessible format, with options ranging from Yes/No answers to multiple-choice and short descriptive answers. Data analysis was performed based on demographics such as age, education, and previous visits to the PAC clinic.

Participant profile

Adult patients (above 18 years) posted for routine surgical procedures and their attendants visiting the PAC clinic, and those who had given their consent, were included in the study. Socioeconomic status, educational background, and urban-rural classification of participants were recorded as part of the demographic data during the PAC clinic registration process. Socioeconomic status was self-reported by the participants and classified using the modified Kuppuswamy scale (2021 update) [5], which considers occupation, education, and monthly family income. Educational status was categorized into five levels: illiterate, primary, secondary, higher secondary, and graduate/postgraduate. Urban or rural residence was determined based on the participant’s registered address and official government classification of residence zones. This classification helped contextualize the findings by exploring how background variables might influence comprehension of pre-anesthesia services. Individuals with hearing problems, unable to speak, and with altered mental status were excluded from the study along with patients refusing to participate. 

Data management and statistical methods

Data was expressed as frequencies and percentages. All categorical data, including demographic distributions and questionnaire responses, were presented in both absolute numbers and percentages for clarity. Correlation studies were performed by unpaired t-test and one-way analysis of variance using IBM SPSS Statistics for Windows, Version 22 (Released 2013; IBM Corp., Armonk, New York, United States) to study the effect of variables like age, sex, literacy level, and previous visit to the PAC clinic on the patient's knowledge and understanding of the PAE. The value of p<0.05 was considered statistically significant. A post-hoc test was applied wherever required using the Tukey’s Honest significant difference (HSD) procedure.

Ethics and consent

An approval was sought from the institutional research cell and the institutional ethics committee of the All India Institute of Medical Sciences Nagpur (Approval number: IEC/Pharmac/2021/194) before the commencement of the study. The anonymity and confidentiality of all the participants was ensured. The participants were informed about the study objectives and the methodology of answering the questionnaires in detail. They were also informed that they had the right to refuse and withdraw at any time. A comprehensive consent form, both in English and the local language (Hindi and Marathi), was provided. 

## Results

Sample characteristics

**Figure 1:**
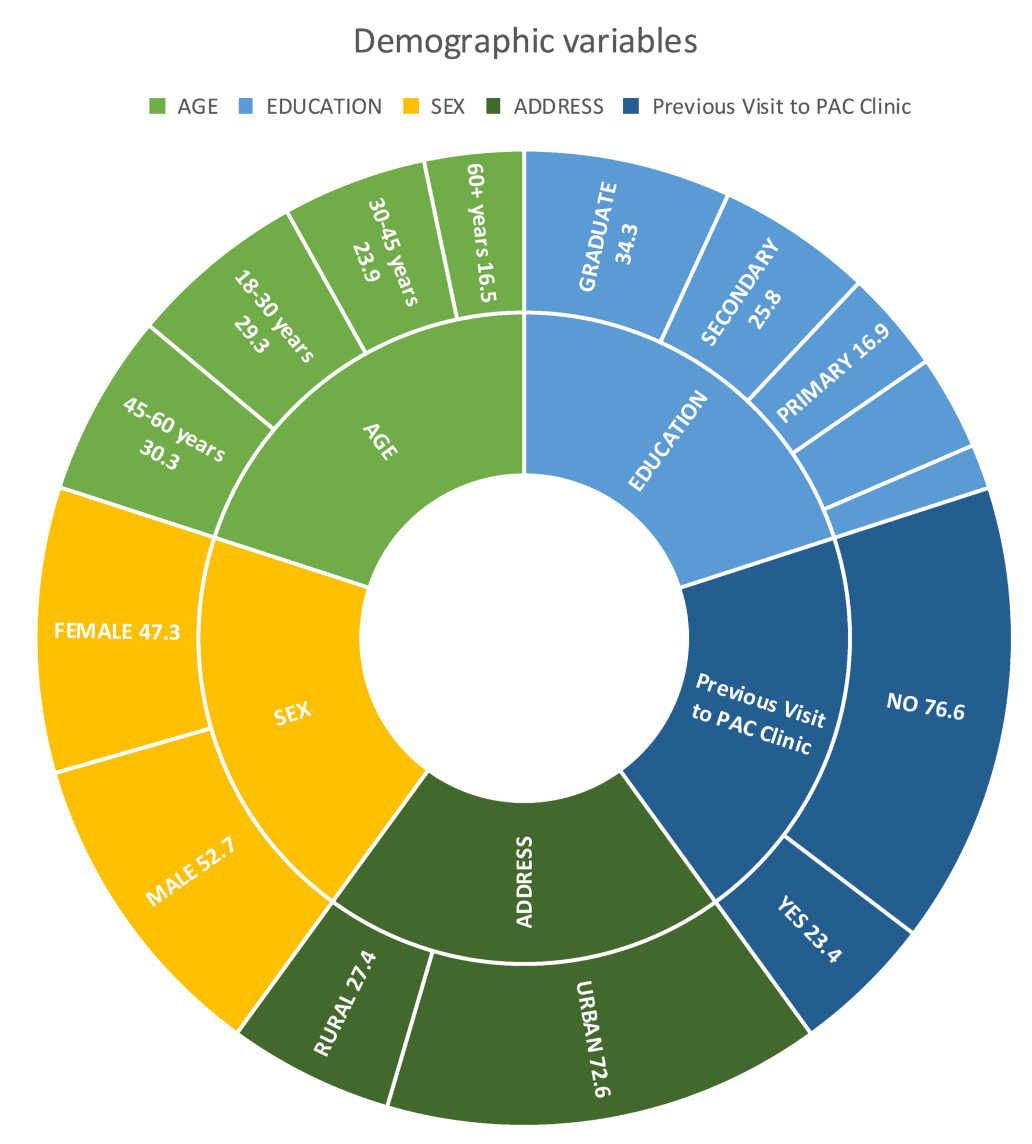
Sunburst chart of demographic variables showing percentage distribution in various categories (n=413)

A total of 413 individuals who visited the PAC clinic gave their consent and answered the questionnaire. Among them, 218 (52.78%) were male and 195 (47.22%) were female subjects. A total of 300 participants (72.64%) belonged to urban residential areas, whereas the remaining 113 (27.36%) were from rural areas. About 97 participants (23.49%) had visited the PAC clinic more than once, and for the remaining 316 (76.6%), it was their first visit. The distribution of patients across different age groups and educational levels is depicted in Figure 1.

Responses to the questionnaire

Of the 413 participants, 124 (30%) correctly understood the purpose of their visit to the PAC clinic, recognizing it as a PAE. However, more than half of the participants believed that the primary purpose of the PAC clinic visit was to obtain a surgical date. In clinical practice, surgical scheduling is often contingent on the outcome of the PAE, which determines the patient's fitness for surgery.

A total of 92 participants (22.3%) correctly identified that assessment and optimization before surgery were performed through steps taken at the PAE. Additionally, 156 participants (37.9%) correctly identified the anesthesiologist as the primary physician responsible for conducting the PAE, although such evaluations may also involve input from other trained healthcare providers.

Regarding the importance of the PAE, 140 participants (34%) correctly understood that it may reduce the overall risk of anesthesia and surgery. More than half, i.e., 237 participants (57.3%) knew that conditions such as heart disease, breathing difficulties, and renal problems should be communicated to the anesthesiologist before surgery. Furthermore, 178 participants (43.1%) recognized that pre-existing medical conditions should be optimized before surgery. The PAE serves as the initiating point for medical optimization, where the anesthesiologist identifies risk factors and refers patients for further investigations or consultations, as needed, to ensure surgical safety.

Nearly 166 respondents (40%) acknowledged that medical conditions may affect the anesthetic and surgical outcomes and formed the largest group. However, they were not the majority, highlighting a significant knowledge gap that calls for enhanced educational efforts. Regarding lifestyle factors, 165 participants (40%) were unaware that habits such as smoking or drinking could affect anesthesia and surgical outcomes. In contrast, 198 (47.9%) correctly identified the impact of these habits, while 50 (12.1%) disagreed.

A majority of participants, 201 (48.6%), mistakenly believed that the PAE is only required for surgeries performed under anesthesia. When asked about when they should address concerns, questions, or fears regarding anesthesia, 147 participants (35.7%) indicated they would discuss these matters with the surgeon in the ward, while 133 (32.1%) correctly identified the PAC clinic as the appropriate setting for such discussions.

Only 92 respondents (22.2%) correctly identified the anesthesiologist as responsible for managing perioperative pain during the intraoperative and immediate postoperative phases. However, this responsibility often shifts post-discharge, where the surgical team generally oversees ongoing pain management in the routine ward or outpatient care settings. This distinction highlights the importance of improving patient understanding of the roles of the different providers across the perioperative timeline. The details of the responses are mentioned in Table 1.

**Table 1:** Patients' responses to the questionnaire PAC, pre-admission anesthesia consultation

S. No.	Questions	Response/ Options available	Total Patients (n=413)	Percentage (%)
1	Why have you come to the anesthesia clinic?	To comply with surgeons’ instructions	50	12.1
		To get a date for surgery	211	51.1
		For pre anesthesia assessment	124	30.0
		I do not know	28	6.8
2	What is done in a pre-anesthesia clinic?	General assessment of the patient before anesthesia	112	27.2
		Some test is to be performed to assess anesthesia fitness	148	35.8
		Assessment and optimization before surgery	92	22.3
		I don’t know	61	14.7
3	Who can perform the pre-anesthesia check-up?	Nurse/Technician in the PAC clinic	42	10.1
		Doctor sitting in the PAC clinic	201	48.7
		Anesthesiologist in the PAC clinic	156	37.9
		Don’t know	14	3.3
4	What is the importance of the pre-anesthesia check-up before surgery?	Reduces the risk of anesthesia and surgery	140	34
		Required to get date for surgery	202	48.8
		Legal documentation	9	2.1
		Don’t know	62	15.1
5	Should conditions like heart disease, breathing difficulties, and renal problems be disclosed before surgery?	Yes	237	57.3
		No	42	10.2
		Not if well controlled	129	31.2
		Don’t know	5	1.3
6	If there is a pre-existing medical condition, does it need to be optimized before surgery?	Yes	178	43.1
		No	76	18.4
		Not required if not related to surgical condition	117	28.3
		Don’t know	42	10.2
7	Do the above mentioned conditions affect the outcome of anesthesia and surgery?	Yes	166	40.2
		No	91	22.1
		Don’t know	156	37.7
8	Do habits like drinking or smoking affect the outcome of anesthesia and surgery?	Yes	198	47.9
		No	50	12.1
		Don’t know	165	40.0
9	Is the pre-anesthesia check-up required only when surgery is to be performed under anesthesia?	Yes	201	48.6
		No	62	15.1
		Don’t know	150	36.3
10	When should you discuss your fear/queries regarding anesthesia (if any)?	In the pre-anesthesia clinic	133	32.1
		In the ward with the surgeon	147	35.7
		In the operation theatre	80	19.3
		Don’t know	53	12.9
11	Will you follow any advice you get at the pre-anesthesia clinic?	Yes, it is for my own good	174	42
		Yes, till the surgery is performed	65	15.8
		Only if surgeon says so	153	37.1
		Don’t know	21	5.1
12	Who will ensure peri-operative pain management?	The surgeon	154	37.3
		Surgeon’s assistant	35	8.5
		Doctor specialized in anesthesia	92	22.2
		The nurse	49	11.9
		Don’t Know	83	20.1

Comparison between the different demographic variables and knowledge scores

Significant associations were observed between knowledge scores and demographic variables such as age, education level, place of residence, and history of previous visits (p<0.05). In contrast, knowledge scores did not differ significantly based on gender. Moreover, the effect of various age groups on knowledge scores was also highly significant (p<0.001). The details of the same are mentioned in Table 2.

**Table 2:** Comparison of knowledge scores amongst various age groups ^#^Tukey Honest significant difference (HSD) post-hoc test. Significant difference amongst each sub-category. 18-30 vs 30-45, p=0.0000; 30-45 vs 45-60, p=0.0021; 30-45 vs 60+, p=0.0000; 45-60 vs 60+, p=0.0276. SD, Standard deviation

Demographic variable	Categories	Participant (n=413; %)	Mean Score±SD	p-value
Age (years)	18-30	121 (29.30)	7.7±1.8	<0.0001^#^
	30-45	99 (23.97)	9±1.6	
	45-60	125 (30.27)	8.2±1.7	
	60+	68 (16.46)	7.5±1.4	

The knowledge scores were highest in the post graduate group, followed by graduates, and those with secondary and then primary education. They were the least in the illiterate participants. The details for the same are mentioned in Table 3.

**Table 3:** Comparison of knowledge scores amongst the various groups based on their education levels ^#^Tukey Honest significant difference (HSD) post-hoc test. Significant difference between each sub-category. Illiterate vs Primary, p=0.0019; Illiterate vs Secondary, p=0.0009; Illiterate vs graduate, p=0.0000; Illiterate vs Post-graduate, p=0.0000; Primary vs graduate, p=0.0000; Primary vs Post-graduate, p=0.0001; Secondary vs Graduate, p=0.0000; Secondary vs Post-graduate, p=0.0000; Graduate vs Post-graduate, p=0.0000. SD, Standard deviation

Demographic variable	Categories	Participants (n=413; %)	Mean Score ± SD	p-value
Education level	Illiterate	30 (7.26)	6.9 ±2	<0.0001^#^
	Primary	70 (16.95)	8.2±1.7	
	Secondary	106 (25.67)	8.2±1.5	
	Graduate	142 (34.38)	8.6±1.7	
	Post-graduate	65 (15.74)	9.4±1.1	

The knowledge scores were comparable between male and female participants. However, when correlated with variables such as residential address and previous visits to the PAC clinic, the knowledge scores demonstrated statistically significant associations (p<0.05). The details for the same are mentioned in Table 4.

**Table 4:** Comparison of knowledge scores between the various demographic variables *p-value<0.05 - Significant SD, standard deviation; PAC, pre-admission anesthesia consultation

Demographic variables	Categories	Participants (n=413; %)	Mean Score ± SD	p-value
Gender	Male	218 (52.78)	8.2 ± 1.7	0.57
	Female	195 (47.22)	8.1 ± 1.9	
Address	Rural	113 (27.36)	7.6 ± 1.7	<0.0001*
	Urban	300 (72.64)	8.8 ± 1.5	
Previous visit to the PAC clinic	Yes	97 (23.49)	9.6 ± 1.2	<0.0001*
	No	316 (76.51)	7.1 ± 1.7	

## Discussion

It is a well-established fact that PAE is an important aspect of patient care. PAE aims to optimize a patient before surgery, to reduce the risk of anesthesia and surgery, and improve outcomes [6]. Furthermore, it provides an opportunity for the patient to discuss any queries or fears regarding anesthesia [7]. Hence, if a patient shows a lack of interest during a PAE or tries to rush through it, the task of anesthesiologists becomes more difficult. This may result in the patient being inadequately optimized before surgery. It is a well-established fact that both the patient's preoperative physical status and surgical procedure affect morbidity and mortality during surgery.

The rise in daycare surgeries, coupled with the increased patient numbers and limited patient awareness about PAEs, has led to insufficient PAEs, escalating morbidity and mortality rates [2,8]. Kluger et al.'s analysis of the Australian Incident Monitoring Study database revealed 478 cases of incorrect pre-operative assessment and 248 cases of inadequate pre-operative preparation out of 6271 reports. Inadequate pre-anesthetic management resulted in a six-fold increase in mortality, with poor communication being a significant contributing factor [9]. Although the PAE can occur the day before surgery, challenges such as inadequate equipment and time constraints in the ward limit its effectiveness. Hence, emphasizing that patients make pre-operative visits to anesthesia clinics is crucial. This approach has proven cost-effective as it reduces inpatient admission expenses [1,2,10].

In the current study, only 124 participants (30%) correctly identified that their visit to the PAC clinic was for a PAE. In contrast, more than half of the study population mistakenly believed that the clinic visit was intended for scheduling a date for their surgery. About 201 respondents (48.6%) believed that a PAE is necessary only for surgeries performed under anesthesia. When asked when they should discuss any fears or questions about anesthesia, 147 participants (35.7%) said they would do so with the surgeon in the ward, while 133 participants (32.1%) correctly indicated that they would bring up their concerns at the PAC clinic.

Approximately 174 participants (42%) said they would follow the advice provided during the PAE. However, when asked who was responsible for managing pain during the perioperative period, 154 participants (37.3%) incorrectly believed it was the surgeon’s role, whereas only 92 participants (22.2%) correctly identified the anesthesiologist as the specialist responsible for it.

In the current study, we found that participants residing in urban areas had higher knowledge scores than those in rural areas, which can be attributed to better comparative exposure and awareness in the cities via various media platforms [11]. Participants who had previous visits to similar PAC clinics had significantly higher scores than those visiting it for the first time. This can be attributed to a better exposure and handling by an anesthesiologist and awareness during the previous visits. Similar results were found in studies conducted by Baaj et al. and Singla et al. [2,12].

Participants who didn’t know how to read and write (illiterate) had significantly lower scores than those who could. The trend showed that the greater the educational qualification, the higher the knowledge. Similar results were also obtained in the studies conducted by Baaj et al. and Singla et al. [2,12]. The current study found a significant difference in the knowledge scores of the participants falling in the different age groups, with the highest scores observed in the age group of 30 to 45 years. These findings suggest that educational attainment and age-related factors, possibly linked to health awareness or prior exposure to healthcare systems, influence the level of understanding regarding the PAE. Studies by Sultan et al. in Pakistan and Amatya et al. in Nepal also found similar results when age and educational status were compared to the knowledge of PAE [13,14].

We found that gender had no significant impact on the perception and comprehension of the PAE in our study. This result is similar to studies conducted by Singla et al., Baaj et al, and Bharadwaj et al. [2,12,15]. On the contrary, a recent study from Nepal found a significant increase in the level of education and an increase in knowledge in male as compared to female participants [14].

PAC clinics are essential to the perioperative care continuum, where anesthesiologists, as perioperative physicians, assess comorbidities, educate patients, and reduce perioperative risk. Evaluating the patient's and attendant's comprehension of PAE services is crucial, as it influences communication, compliance, and preparedness. Patients expect anesthesiologists to introduce themselves, provide clear information about anesthesia, postoperative care, and pain management, and dedicate sufficient time during the evaluation [1,16]. Patient satisfaction with the PAC clinic visit is strongly tied to these expectations. However, as noted by Gebremedhn et al., only 65% of patients reported satisfaction. This falls short of the standards set by the Royal College of Anaesthetists, highlighting the need to enhance the quality of pre-anesthesia interactions [17].

This study focused exclusively on patients attending in-person PAC clinics and evaluated their understanding and awareness of the PAE process during physical consultations at a tertiary care teaching hospital. The limited awareness and knowledge about PAEs among the general population, can be attributed to several factors. Inadequate public education campaigns and limited access to healthcare facilities contribute to a lack of information dissemination [18,19]. Low health literacy levels, cultural beliefs, and stigma surrounding medical procedures can discourage individuals from seeking information. Insufficient communication between healthcare professionals and patients, as well as an overemphasis on the surgical aspects rather than the PAE, further hampers awareness [19,20]. Additionally, the digital divide and the complacency among patients who have not faced adverse events during surgeries also play a role in the poor understanding of PAEs [21]. Addressing these challenges requires comprehensive efforts, including targeted awareness campaigns, improved physician-patient communication, culturally sensitive education, and initiatives to bridge the digital gap, ensuring that accurate and accessible information about PAEs reaches all segments of the population.

Enhancing patients' awareness and understanding of PAEs is crucial for ensuring their safety and the success of medical procedures. Healthcare providers and institutions can take several measures to achieve this goal. Firstly, clear communication is essential, involving the use of simple language and providing written materials explaining PAEs. Additionally, creating specific educational materials or videos, conducting interactive sessions, and utilizing digital resources such as user-friendly websites and mobile apps can significantly enhance patient education [22]. Providing multilingual materials, engaging family members, and using visual aids like diagrams can simplify complex medical information. Follow-up calls, patient portals, and feedback mechanisms also contribute to ongoing patient education. Continuous training for healthcare providers in effective communication techniques is essential, as well as community outreach programs to educate the general public about the significance of PAEs. By implementing these strategies, healthcare providers can empower patients with knowledge, leading to improved understanding, compliance, and overall healthcare outcomes.

There is an urgent requirement for national guidelines tailored to the disease profile of Indian patients, involving anesthesia and surgery professionals as key stakeholders. These guidelines should ideally be standardized across healthcare facilities in India, including rural and compromised setups in semi-urban and lower-tier cities. Implementing electronic pre-anesthesia forms can enhance data quality, meet anesthesiologists’ expectations, and significantly reduce the risk of missing crucial preoperative information, ensuring the safety of the patients [23]. Effective communication and documentation play a pivotal role in preoperative risk assessment. Standardized documentation methods can address perioperative risks and disease-related management issues comprehensively, enhancing communication with surgical colleagues, minimizing unnecessary delays, and reducing complications, especially in high-risk patients. A systematic risk assessment approach, coupled with disease-specific recommendations, can greatly contribute to achieving these goals [24]. While our study focused primarily on assessing the comprehension and awareness of PAE services among patients and their attendants, we recognize that evaluating patient satisfaction is another important indicator of its effectiveness. Although satisfaction was not within the scope of our current objectives, incorporating structured satisfaction scoring in future studies could provide a more holistic understanding of patient experiences in PAC clinic settings. This would complement the current findings by linking comprehension levels with perceived quality of care.

Strengths

The study's strength lies in its detailed analysis of several demographic variables, revealing the knowledge disparities. It identifies specific gaps, enabling targeted educational interventions. Additionally, its relevance in the pandemic era highlights the need to address these gaps, especially with the rise of telemedicine and virtual healthcare practices. This valuable insight informs strategies for enhancing patient awareness effectively.

Limitations

The study's limitations include its single-center design, which may limit the generalizability of the findings to other regions or populations. The sample size, while adequate, may not fully represent the diversity of patients across various settings. Additionally, the questionnaire does not delve deeply into the specific concerns of the patients, and temporal factors, such as changing healthcare policies during the pandemic, were not considered. Furthermore, although the survey was anonymous, potential biases in patient responses remain, especially if certain questions were unclear or sensitive. Finally, significant urban-rural disparities in healthcare access and education may affect the knowledge of PAEs, suggesting that future studies should explore these factors further.

Despite these limitations, the study provides valuable insights into patients' knowledge of PAEs, but the broader implications should be considered cautiously. Future multi-center studies with larger sample sizes and more comprehensive questionnaires could help address these limitations and improve the generalizability of the findings.

## Conclusions

This study illuminates the gaps in patient awareness regarding PAEs, a critical component of safe surgical procedures. Despite the pivotal role of anesthesiologists, patients exhibit varying degrees of understanding, often misconstruing the purpose of PAC clinics. The findings underscore the necessity for intensified educational efforts and comprehensive communication strategies to bridge this knowledge divide. Importantly, the demographic disparities further highlight the need for targeted interventions, especially among rural and less-educated populations. The study emphasizes the vital role of clear, accessible, and culturally sensitive patient education, supported by ongoing healthcare provider training, to enhance patient comprehension and engagement, thereby promoting safer surgical practices.
